# The *Tetramorium tortuosum* species group (Hymenoptera, Formicidae, Myrmicinae) revisited - taxonomic revision of the Afrotropical *T. capillosum* species complex

**DOI:** 10.3897/zookeys.299.5063

**Published:** 2013-05-14

**Authors:** Francisco Hita Garcia, Brian L. Fisher

**Affiliations:** 1 Entomology, California Academy of Sciences, 55 Music Concourse Drive, San Francisco, CA 94118, U.S.A.

**Keywords:** Afrotropical region, Central Africa, equatorial rainforests, taxonomy, Tetramoriini, *Tetramorium*, *Tetramorium capillosum* species complex, *Tetramorium tortuosum* species group

## Abstract

In this study we revise the taxonomy of the *Tetramorium tortuosum* species group members encountered in the Afrotropical region, which we have placed in its own subgroup: the *Tetramorium capillosum* species complex. We re-describe the two previously known species *Tetramorium capillosum* Bolton and *Tetramorium tabarum* Bolton, and describe the new species *Tetramorium hecate* **sp. n.** The geographic distribution of the three species appears to be restricted to the equatorial rainforests of Central Africa. We provide a diagnosis of the *Tetramorium capillosum* species complex, an illustrated identification key to species level, and worker-based species descriptions, which include diagnoses, discussions, high-quality montage images, and distribution maps. Furthermore, we discuss biogeography and composition of the globally distributed *Tetramorium tortuosum* group.

## Introduction

With approximately 500 valid species (Bolton 2012, Hita Garcia and Fisher 2012a, 2012b), the genus *Tetramorium* Mayr represents one of the most species-rich ant genera, a group that is also widely distributed throughout most zoogeographical regions. However, in terms of species and species group numbers, the main diversity of the genus is found in the Afrotropical and Malagasy regions, from where around 220 Afrotropical and 84 Malagasy species are currently known (Bolton 1976, 1979, 1980, 1985, Hita Garcia et al. 2010a, Hita Garcia et al. 2010b, 2010c, Hita Garcia and Fisher 2011, 2012a, 2012b). The majority of the remaining described species diversity (ca. 190 species) is distributed in the Palaearctic, Oriental, and Indo-Australian regions. By contrast, the *Tetramorium* fauna of the New World is rather depauperate with only seven native species (Bolton 1977, Marques et al. 2011, Vásquez-Bolaños 2007, Vásquez-Bolaños et al. 2011).

Despite its large number of species, wide distribution, and relatively high abundance in many regions, the taxonomy of the genus is in a moderately good condition, mainly due to Bolton’s (1976, 1977, 1979, 1980, 1985) revisionary studies. Of special importance was his treatment of the tribe Tetramoriini (Bolton 1976), in which he recognised the genera *Atopula* Emery, *Macromischoides* Wheeler, *Tetrogmus* Roger, and *Xiphomyrmex* Forel to be junior synonyms of *Tetramorium*, as well as the later synonymisation of *Triglyphothrix* Forel under *Tetramorium* (Bolton 1985). These studies clearly defined the genus *Tetramorium* and separated it from the other genera in the tribe. Prior to Bolton’s work, the definitions of the tribe and its constituent genera were relatively vague and changed regularly depending on whether authorities treated the tribe partly or in its entirety (Bolton 1976). However, most modern ant sampling projects in the Afrotropical region (Belshaw and Bolton 1993, 1994, Braet and Taylor 2008, Deblauwe and Dekoninck 2007, Dejean et al. 2000, Fisher 2004, Hita Garcia et al. 2009, Jackson 1984, Robertson 1999, 2002, Watt et al. 2002, Yanoviak et al. 2007, Yeo et al. 2011) have been carried out in the decades after Bolton’s studies. These surveys yielded much new material that resists identification using the keys of Bolton (1976, 1980). Consequently, despite having a good taxonomic foundation, most Afrotropical species groups would benefit from updated taxonomic revisions that incorporate material generated after 1980. Recently, this was done for the relatively species-rich *Tetramorium weitzeckeri* species group (Hita Garcia et al. 2010b, 2010c), but all other groups still await taxonomic treatments.

The *Tetramorium tortuosum* species group is distributed throughout the Old and New World tropics and subtropics, and with currently 50 species is one of the most species-rich groups within the genus (Bolton 1977, 1979, 1980, Marques et al. 2011, Vásquez-Bolaños 2007, Vásquez-Bolaños et al. 2011). As noted above, the New World has only seven native species all belonging to the *Tetramorium tortuosum* group (Bolton 1977, Marques et al. 2011, Vásquez-Bolaños 2007, Vásquez-Bolaños et al. 2011). The group is also well-represented in the Oriental and Indo-Australian regions with eight and ten species respectively (Bolton 1977, Sheela and Narendran 1998). Interestingly, the group attains its highest species richness in the Malagasy region, where 22 species are known (Hita Garcia and Fisher 2012b). Despite having the highest diversity of species and species groups within the genus *Tetramorium*, the Afrotropical region harbours only three *Tetramorium tortuosum* group species, which seems very low compared to the other regions as already noted by Bolton (1980).

The taxonomic foundation for the species group is in relatively good condition. The species from the Oriental and Indo-Australian regions were revised by Bolton (1977) and his keys still work relatively well, although it is not unlikely that they might have to be updated in future revisions of the regions that incorporate material sampled after 1977. The seven New World species can be identified with the latest key from Vásquez-Bolaños et al. (2011), and Hita Garcia and Fisher (2012b) provide identification keys for the species-rich Malagasy fauna in their recent revision. In this study we revise the taxonomy of the *Tetramorium tortuosum* group species encountered in the Afrotropical region, which we have placed in its own subgroup: the *Tetramorium capillosum* species complex. We describe the two previously known species, *Tetramorium capillosum* Bolton and *Tetramorium tabarum* Bolton, and describe *Tetramorium hecate* sp. n. as new species. All descriptions include high-quality montage images and distribution maps. Furthermore, an illustrated identification key for the Afrotropical region is provided, and the biogeography and composition of the *Tetramorium tortuosum* species group is discussed.

## Abbreviations of depositories

The collection abbreviations follow Bolton (1980) and Evenhuis (2009). The material upon which this study is based is located and/or was examined at the following institutions:

**BMNH** The Natural History Museum (British Museum, Natural History), London, U.K.

**CASC** California Academy of Sciences, San Francisco, California, U.S.A.

**MCZ** Museum of Comparative Zoology, Cambridge, Massachusetts, U.S.A.

**MHNG** Muséum d’Histoire Naturelle de la Ville de Genève, Geneva, Switzerland

**NHMB** Naturhistorisches Museum, Basel, Switzerland

## Material and methods

The material examined in this study is located in the collections of BMNH, CASC, and MCZ. More than 95% of the specimens examined belong to CASC and were sampled during ant inventories carried out in Central Africa from 1998 to 2001 (Fisher 2004, B.L.F., unpublished data), whereas the type series of *Tetramorium capillosum* and *Tetramorium tabarum*, together with a small amount of non-type specimens, are found in BMNH and MHNG.

All new type material and all imaged specimens can be uniquely identified with specimen-level codes affixed to each pin (e.g. CASENT0078328). In the presented descriptions we list all of the available specimen-level codes for the whole type series. It should be noted, however, that the number of stated paratype workers does not necessarily match the number of listed specimen-level codes because pins can sometimes hold more than one specimen. Digital colour images were created using a Leica DFC 425 camera in combination with the Leica Application Suite software (version 3.8). All images presented are available online and can be seen on AntWeb (http://www.antweb.org). The measurements were taken with a Leica MZ 12.5 equipped with an orthogonal pair of micrometers at a magnification of 100×, rarely 80×. Measurements and indices are presented as minimum and maximum values with arithmetic means in parentheses. In addition, all measurements are expressed in mm to two decimal places. The measurements and indices used in this study are the same as in Hita Garcia and Fisher (2011, 2012a, 2012b):

Head length (HL): maximum distance from the mid-point of the anterior clypeal margin to the mid-point of the posterior margin of head, measured in full-face view. Impressions on anterior clypeal margin and posterior head margin reduce head length.

Head width (HW): width of head directly behind the eyes measured in full-face view.

Scape length (SL): maximum scape length excluding basal condyle and neck.

Eye length (EL): maximum diameter of compound eye measured in oblique lateral view.

Pronotal width (PW): maximum width of pronotum measured in dorsal view.

Weber’s length (WL): diagonal length of mesosoma in lateral view from the postero-ventral margin of propodeal lobe to the anterior-most point of pronotal slope, excluding the neck.

Propodeal spine length (PSL): the tip of the measured spine, its base, and the centre of the propodeal concavity between the spines must all be in focus. Using a dual-axis micrometer the spine length is measured from the tip of the spine to a virtual point at its base where the spine axis meets orthogonally with a line leading to the median point of the concavity.

Petiolar node height (PTH): maximum height of petiolar node measured in lateral view from the highest (median) point of the node to the ventral outline. The measuring line is placed at an orthogonal angle to the ventral outline of the node.

Petiolar node length (PTL): maximum length of the dorsal face of the petiolar node from the anterodorsal to the posterodorsal angle, measured in dorsal view excluding the peduncle.

Petiolar node width (PTW): maximum width of dorsal face of petiolar node measured in dorsal view.

Postpetiole height (PPH): maximum height of the postpetiole measured in lateral view from the highest (median) point of the node to the ventral outline. The measuring line is placed at an orthogonal angle to the ventral outline of the node.

Postpetiole length (PPL): maximum length of postpetiole measured in dorsal view.

Postpetiole width (PPW): maximum width of postpetiole measured in dorsal view.

Ocular index (OI): EL / HW * 100

Cephalic index (CI): HW / HL * 100

Scape index (SI): SL / HW * 100

Propodeal spine index (PSLI): PSL / HL * 100

Petiolar node index (PeNI): PTW / PW * 100

Lateral petiole index (LPeI): PTL / PTH * 100

Dorsal petiole index (DPeI): PTW / PTL * 100

Postpetiolar node index (PpNI): PPW / PW * 100

Lateral postpetiole index (LPpI): PPL / PPH * 100

Dorsal postpetiole index (DPpI): PPW / PPL * 100

Postpetiole index (PPI): PPW / PTW * 100

Pubescence and pilosity are often of high diagnostic value within the genus *Tetramorium* (Bolton 1976, 1977, 1979, 1980, 1985, Hita Garcia et al. 2010b, Hita Garcia and Fisher 2011, 2012a, 2012b). The varying degree of inclination of pilosity is particularly important for the diagnosis of groups or species. In this context we use the terms “erect”, “suberect”, “subdecumbent”, “decumbent”, and “appressed” following Wilson (1955).

## Results

### Synopsis of Afrotropical *Tetramorium tortuosum* species group

***Tetramorium capillosumspecies complex***

*Tetramorium capillosum* Bolton, 1980

*Tetramorium hecate* Hita Garcia & Fisher **sp. n.**

*Tetramorium tabarum* Bolton, 1980

**Diagnosis of Afrotropical *Tetramorium capillosum* species complex**

Eleven-segmented antennae; antennal scape short to moderately long (SI 73 - 86); anterior clypeal margin usually entire without median notch; frontal carinae very well developed and usually reaching posterior head margin; antennal scrobe present, weakly to very well developed; propodeal spines medium-sized to long, elongate-triangular to spinose; propodeal lobes short, triangular to elongate-triangular; petiolar node in profile nodiform, in profile as high as long to 1.3 times higher than long (LPeI 78 - 100), in dorsal view always longer than wide (DPeI 80 - 93); postpetiole sub-globular to moderately anteroposteriorly compressed; mandibular sculpture variable; cephalic sculpturation distinct, between frontal carinae longitudinally rugose to reticulate-rugose; mesosoma predominantly longitudinally rugose; petiolar node weakly to distinctly rugose, postpetiole ranging from unsculptured to longitudinally rugose; gaster unsculptured, smooth, and shiny; all dorsal surfaces of body with abundant, long, standing hairs; first gastral tergite without pubescence, and pilosity never short, dense, and appressed; sting appendage spatulate.

**Taxonomic notes**

In the Afrotropical region, members of the *Tetramorium tortuosum* group are unlikely to be misidentified with species from the other three groups having 11-segmented antennae. The 26 species of the *Tetramorium weitzeckeri* group all have a squamiform or high nodiform petiolar node, which is always significantly wider than long. This node shape strongly contrasts with the shape observed in the *Tetramorium tortuosum* group since all three members have a nodiform node which is much longer than wide. The second species group, the *Tetramorium angulinode* group, is morphologically closer to the *Tetramorium tortuosum* group since both groups share a nodiform petiolar node. However, they can be clearly separated by the pilosity/pubescence patterns on the first gastral tergite. In the *Tetramorium tortuosum* group pubescence is absent and pilosity is long and mainly erect, whereas in the *Tetramorium angulinode* group pubescence and pilosity are usually present, dense, appressed to decumbent, and often pointed towards a longitudinal midline of the tergite. The synonymisation of *Triglyphothrix* under *Tetramorium* (Bolton 1985) added an additional group, the *Tetramorium ericae* group, with few species that possess 11-segmented antennae. However, these species are all comparatively small and have branched pilosity on most of the body, thus not easily confused with the much larger *Tetramorium tortuosum* group species that all possess simple pilosity.

The taxonomy of the *Tetramorium tortuosum* group in the Malagasy region can be challenging, and species delimitations for several group members proved difficult (Hita Garcia and Fisher 2012b). However, this was not the case for the three Afrotropical species. *Tetramorium capillosum*, *Tetramorium hecate*, and *Tetramorium tabarum* are very easy to distinguish, and the species delimitations presented in this study are straightforward and transparent. This is partly due to the small number of species in Africa. However, the three species are also often found in sympatry, and the fact that they maintain their species-specific characteristics without any intermediate forms provides further evidence for their heterospecificity.

Hita Garcia and Fisher (2012b) introduced four species complexes for the *Tetramorium tortuosum* group in the Malagasy region (*Tetramorium andrei*, *Tetramorium jedi*, *Tetramorium noeli*, and *Tetramorium smaug* complexes), and it seems appropriate to evaluate whether the Afrotropical species fit into one of these groups or deserve their own species complex. Based on the definitions of the complexes, the Afrotropical species cannot be members of the *Tetramorium jedi*, *Tetramorium noeli*, or *Tetramorium smaug* complexes due to a lack of sculpture on the forecoxae and the first gastral tergite. This would argue for placement in the *Tetramorium andrei* complex. Two reasons prevented us from doing so however. First, there is a difference in the development of the anterior clypeal margin, which is strongly medially impressed in the Malagasy *Tetramorium andrei* complex while the Afrotropical species treated in this study usually have an entire margin (except for some specimens of *Tetramorium hecate* that possess a very small notch that is challenging to see without higher magnifications). The second reason not to place the African species into a Malagasy complex is the current uncertainty about whether the *Tetramorium tortuosum* group as a whole is a natural group of closely related species, an issue discussed below. Consequently, we propose to place *Tetramorium capillosum*, *Tetramorium hecate*, and *Tetramorium tabarum* in their own complex, the *Tetramorium capillosum* species complex.

**Biogeographic notes on the group**

The three Afrotropical species of the *Tetramorium tortuosum* group have a moderately restricted distribution range since they are only known from Equatorial rainforests in the Central African countries of Gabon, Cameroon, Democratic Republic of Congo, Central African Republic, and Uganda. Given all of the known African localities, the distribution of *Tetramorium capillosum* and *Tetramorium tabarum* appears fairly disjunctive. Most localities are located in the west of the distribution range in Gabon, Cameroon, and western parts of the Central African Republic, but few localities are found much further east in the northeastern Democratic Republic of Congo and northwestern Uganda. This represents a great gap between these two groups of localities. However, we think that this lack of occurrence records is very likely due to a sampling artefact since ant sampling has been relatively fragmentary in sub-Saharan Africa. The westernmost known distribution limit is the eastern coast of the Gulf of Guinea and the easternmost known locality appears to be the Budongo Forest in northwestern Uganda. It is unlikely that they occur further east, which is supported by an inventory of the myrmecofauna of the Kakamega Forest in Western Kenya (Hita Garcia et al. 2009). The latter study yielded 40 species of *Tetramorium* in this rainforest locality but no member of the *Tetramorium tortuosum* group could be collected, even though the sampling effort was comparatively high. The same is true for West Africa. Several ant sampling projects were carried out northwest of the known distribution range of the group; e.g. in Ghana (Belshaw and Bolton 1993, 1994, Majer 1972, 1976a, 1976b, Room 1971), Nigeria (Taylor 1977, 1978), or Ivory Coast (Kone et al. 2012, Yeo et al. 2011). However, none of these projects collected a single *Tetramorium tortuosum* group member. Consequently, it is safe to say that the Afrotropical members of the group are all restricted to the Equatorial rainforests of Central Africa.

As mentioned above, the *Tetramorium tortuosum* species group is remarkably species-poor in the Afrotropical region. Bolton (1980) provided two alternative scenarios to explain why the group fauna is so depauperate in Africa. One postulate suggests that the group originated elsewhere outside Africa and colonized the continent relatively late, when it faced strong competition from other *Tetramorium* groups that had already occupied the niches of *Tetramorium tortuosum* group members. The second hypothesis presented by Bolton (1980) postulates that the group was once as diverse in the Afrotropical region as elsewhere, but has been displaced by recently developed and possibly better-adapted *Tetramorium* competitors, namely the *Tetramorium weitzeckeri* group species. Bolton (1980) preferred the second hypothesis, and even though little more evidence exists today than in 1980, we concur with him. Indeed, several medium-sized to large species of the *Tetramorium weitzeckeri* group, such as *Tetramorium boltoni* Hita Garcia, Fischer and Peters, *Tetramorium guineense* (Bernard), *Tetramorium philippwagneri* Hita Garcia, Fischer and Peters, and *Tetramorium pinnipilum* Bolton, are fairly successful, common, and relatively abundant species found in many equatorial rainforests. Also, the *Tetramorium weitzeckeri* group, with the exception of *Tetramorium humbloti* Forel that has invaded the Malagasy region, is restricted in distribution to the Afrotropical region. This fact indicates that it represents a relatively young and successful development within the Afrotropical *Tetramorium* fauna, supporting Bolton’s (1980) hypothesis. Furthermore, apart from the strong competition from the *Tetramorium weitzeckeri* group, there are many more Afrotropical species groups with 12-segmented antennae that perform well in rainforests, such as the *Tetramorium bicarinatum*, *Tetramorium camerunense*, and *Tetramorium flabellum* groups. This very strong competition in most modern-day rainforests might well explain the depauperate *Tetramorium tortuosum* fauna encountered in Africa. The species-rich Malagasy group fauna does not face the same competition by other genus members within their size and niche ranges, and with 22 species seems to have undergone a fairly successful radiation, mostly in the rainforests of eastern Madagascar (Hita Garcia and Fisher 2012b). However, it remains unclear whether the three Afrotropical *Tetramorium tortuosum* group species are a relic of a formerly more successful group fauna or the species group colonised the African continent relatively late.

A different scenario not mentioned by Bolton (1980) offers a third theory. In the recent revision of the Malagasy group fauna Hita Garcia and Fisher (2012b) discuss the possibility that the current *Tetramorium tortuosum* group might not be a natural group in a phylogenetic sense. There is a great deal of variation within the group, especially in the Oriental and Indo-Australian regions, and the few key characters that distinguish the group might have evolved several times independently in different zoogeographic regions. If true, then the Afrotropical group fauna would be an isolated Afrotropical development not closely related to the other group members from the New World, the Malagasy, Oriental, and Indo-Australian regions. Hita Garcia and Fisher (2012b) point out that it would be possible to split the *Tetramorium tortuosum* group under its current definition into several regional groups, but are reluctant to do so without further evidence from other regions than the Malagasy. Bearing all this in mind, we remain hesitant to split the *Tetramorium tortuosum* group into several regional species groups, even though there might be characters to support this action for the species from the New World, the Afrotropical and Malagasy regions (Hita Garcia and Fisher 2012b). The situation for the remainder of the species from the Oriental and Indo-Australian regions is murkier, however. In our opinion it is only possible to assess the validity of the *Tetramorium tortuosum* group within the framework of a larger-scale molecular phylogenetic analysis of the genus *Tetramorium* including most species groups and all regions. This might reveal evidence for a reorganisation of the *Tetramorium tortuosum* group under its current definition, but until then the species treated in this study must be considered authentic members of the group.

### Identification key for Afrotropical *Tetramorium capillosum* species complex (workers)

**Table:** 

1	Antennal scapes relatively short (SI 73 - 77); petiolar node in profile clearly rectangular nodiform with anterodorsal and posterodorsal margins at about same height (Fig. 1A)	*Tetramorium hecate*
–	Antennal scapes always longer (SI 80 - 86); petiolar node nodiform with less rectangular and more rounded margins, anterodorsal margin situated lower than posterodorsal margin (Figs 1B, C)	2
2	Mandibles strongly longitudinally sculptured; eyes moderately large (OI 23 - 25); whole body dark brown to black in colour (Figs 2A, C)	*Tetramorium capillosum*
–	Mandibles unsculptured, smooth and shiny; eyes very large (OI 27 - 31); body bicoloured, head, mesosoma, waist segments yellow to bright orange, gaster very dark brown to black (Figs 2B, D)	*Tetramorium tabarum*

#### Tetramorium capillosum

Bolton

http://species-id.net/wiki/Tetramorium_capillosum

**Figure 1.**
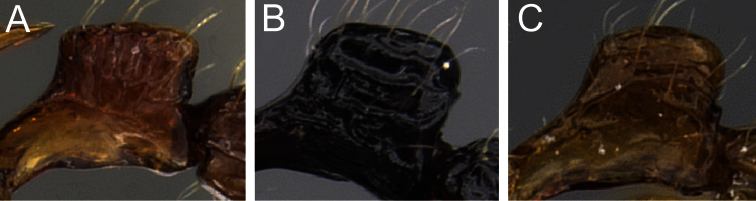
Petiolar node in profile. **A** *Tetramorium hecate* (CASENT0248334) **B** *Tetramorium capillosum* (CASENT0901156) **C** *Tetramorium tabarum* (CASENT0280900).

**Figure 2.**
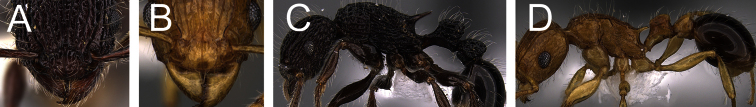
**A, B** Head in full-face view. **A** *Tetramorium capillosum* (CASENT0901156) **B** *Tetramorium tabarum* (CASENT0316967) **C, D** Body in profile. **C** *Tetramorium capillosum* (CASENT0316960) **D** *Tetramorium tabarum* (CASENT0316967).

**Figure 3.**
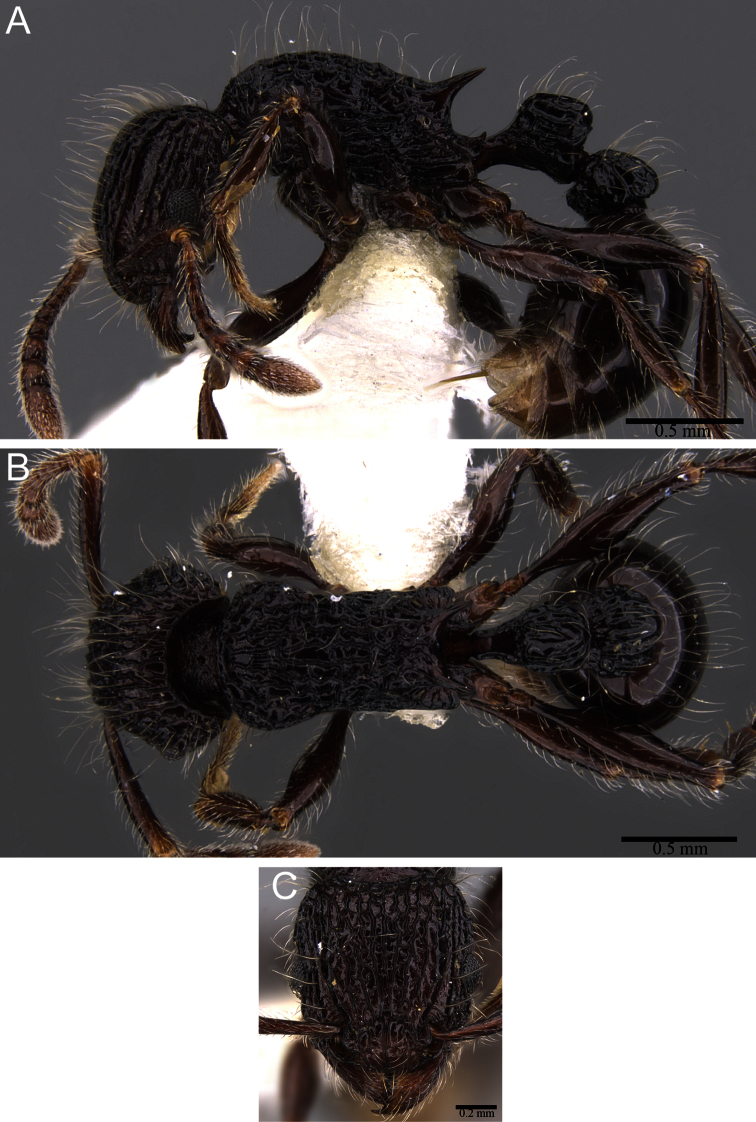
*Tetramorium capillosum* paratype worker (CASENT0901156). **A** Body in profile **B** Body in dorsal view **C** head in full-face view.

Figures 1B , 2A, 2C , 3A, 3B, 3C , 7

Tetramorium capillosum Bolton, 1980: 236.

##### Type material.

Holotype, pinned worker, GABON, Makokou, 0°34'N, 12°52'E, rainforest, X.1972 (*I. Lieberburg*) [MCZ] [examined]. Paratypes, seven pinned workers with same data as holotype [BMNH; MCZ] [examined].

**Non-type material.** CAMEROON: Mbalmayo, XI.1993 (*N. Storck*); Ndupe, 20.XII.1989 (*A. Dejean*); Sud, Bondé Forest, N’kolo village, 27.5 km 155° SSE Elogbatindi, 3.2217N, 10.2467E, 40 m, rainforest, 12.IV.2000 (*B.L. Fisher*); Sud, Campo Reserve, 2°36'N, 9°56'E, 40 m, 25.X.1991 (*D.M. Olson*); Sud, P.N. Campo, 43.3 km 108°ESE Campo, 2.2825N, 10.2062E, 290 m, rainforest, 7.IV.2000 (*B.L. Fisher*); Sud, Res. de Faune de Campo, Massif des Mamelles, 15.1 km 84°E Ébodjé, 2.59417N, 9.9595E, 180 m, rainforest, 4.IV.2000 (*B.L. Fisher*); CENTRAL AFRICAN REPUBLIC: Prefecture Sangha-Mbaéré, Réserve Spéciale de Forêt Dense de Dzanga-Sangha, 12.7 km 326°NW Bayanga, 3.005N, 16.1933E, 420 m, rainforest, 10.–17.V.2001 (*B.L. Fisher*); Prefecture Sangha-Mbaéré, Parc National Dzanga-Ndoki, Mabéa Bai, 21.4 km 53°NE Bayanga, 3.0333N, 16.41E, 510 m, rainforest, 1.–7.V.2001 (*B.L. Fisher*); DEMOCRATIC REPUBLIC OF CONGO: Epulu, 1.3833N, 28.5833E, 750 m, rainforest, 1.XI.1995 (*S.D. Torti*); GABON: La Makande, Foret de Abeilles, I.-II.1999 (*S. Lewis*); Makokou, rainforest, X.1972 (*I. Lieberburg*); Ogooue-Maritime, Aire d’Exploit. Rationnelle de Faune des Monts Doudou, 24.3 km 307°NW Doussala, 2.2264N, 10.4097E, 375 m, rainforest, 6.-9.III.2000 (*B.L. Fisher*); Ogooue-Maritime, Aire d’Exploit. Rationnelle de Faune des Monts Doudou, 25.2 km 304°NW Doussala, 2.2275S, 10.3945E, 640 m, rainforest, 14.III.2000 (*B.L. Fisher*); Ogooue-Maritime, Reserve des Monts Doudou, 25.2 km 304°NW Doussala, 2.2272S, 10.3945E, 630 m, coastal lowland rainforest, 13.-20.III.2000 (*S. van Noort*); Ogooue-Maritime, Reserve de la Moukalaba-Dougoua, 12.2 km 305°NW Doussala, 2.3167S, 10.5333E, 110 m, rainforest, 24.II.2000 (*B.L. Fisher*); Ogooue-Maritime, Reserve de la Moukalaba-Dougoua, 10.8 km 214°SW Doussala, 2.4227S, 10.5453E, 110 m, rainforest, 29.II.2000 (*B.L. Fisher*); Ogooue-Maritime, Reserve de la Moukalaba-Dougoua, 12.2 km 305°NW Doussala, 2.2833S, 10.4972E, 110 m, coastal lowland rainforest, 24.II.–3.III.2000 (*S. van Noort*); Woleu-Ntem, 31.3 km 108°ESE Minvoul, 2.08N, 12.4067E, 600 m, rainforest, 7.II.1998 (*B.L. Fisher*); UGANDA: Bunyoro District, Budongo Forest FS, 1.7264N, 31.5524E, 1081 m, 8.VII.2009 (*W. Freund & T. Klug*).

##### Diagnosis.

The following character combination clearly distinguishes *Tetramorium capillosum* from the remainder of the species group: eyes of moderate size (OI 23 - 25); antennal scapes moderately long (SI 80 - 83); petiolar node nodiform with anterodorsal and posterodorsal margins relatively rounded, posterodorsal margin situated higher than anterodorsal margin, dorsum convex; mandibles strongly longitudinally rugose; petiole and postpetiole usually with weak sculpture; whole body uniformly very dark brown to black.

##### Worker measurements

**(N=12).** HL 0.79 - 0.89 (0.84); HW 0.76 - 0.84 (0.79); SL 0.62 - 0.69 (0.65); EL 0.18 - 0.21 (0.19); PH 0.41 - 0.51 (0.46); PW 0.60 - 0.68 (0.65); WL 1.02 - 1.19 (1.12); PSL 0.26 - 0.38 (0.30); PTL 0.31 - 0.37 (0.34); PTH 0.34 - 0.41 (0.37); PTW 0.28 - 0.33 (0.31); PPL 0.28 - 0.33 (0.30); PPH 0.37 - 0.43 (0.40); PPW 0.37 - 0.44 (0.41); CI 94 - 96 (95); SI 80 - 83 (82); OI 23 - 25 (24); DMI 55 - 62 (58); LMI 39 - 43 (41); PSLI 31 - 43 (35); PeNI 45 - 49 (47); LPeI 89 - 100 (94); DPeI 86 - 94 (89); PpNI 61 - 66 (63); LPpI 70 - 78 (75); DPpI 130 - 139 (135); PPI 127 - 138 (134).

##### Worker description.

Head longer than wide (CI 94 - 96); posterior head margin moderately concave. Anterior clypeal margin entire and convex. Frontal carinae strongly developed, approaching or ending at posterior head margin. Antennal scrobes narrow but very well-developed with clearly defined margins all around. Antennal scapes moderately long, not reaching posterior head margin (SI 80 - 83). Eyes of moderate size (OI 23 - 25). Mesosomal outline in profile weakly convex, moderately marginate from lateral to dorsal mesosoma; promesonotal suture and metanotal groove absent; mesosoma comparatively stout and high (LMI 39 - 43). Propodeal spines relatively long to very long, spinose, and acute (PSLI 31 - 43); propodeal lobes short, triangular to elongate-triangular, and usually acute. Petiolar node in profile rectangular nodiform, approximately as high as long to weakly higher than long (LPeI 89 - 100), anterior and posterior faces approximately parallel, posterodorsal margin situated higher than anterodorsal, anterodorsal and posterodorsal angles relatively rounded, petiolar dorsum convex; node in dorsal view approximately 1.1 times longer than wide  (DPeI 86 - 94). Postpetiole in profile relatively high and moderately anteroposteriorly compressed, approximately 1.2 to 1.3 times higher than long (LPpI 70 - 78); in dorsal view around 1.3 to 1.4 times wider than long (DPpI 130 - 139). Postpetiole in profile thinner and higher than petiolar node, in dorsal view approximately 1.3 to 1.4 times wider than petiolar node (PPI 127 - 138). Mandibles strongly longitudinally rugose; clypeus longitudinally rugulose, usually with three to five rugulae, median rugula better developed; cephalic dorsum between frontal carinae irregularly longitudinally rugose to reticulate rugose, posteriorly towards posterior head margin fully reticulate-rugose, anteriorly towards posterior clypeal margin more regularly longitudinally rugose; scrobal area unsculptured, smooth, and shining; lateral and ventral head longitudinally rugose to reticulate-rugose. Mesosoma laterally and dorsally strongly irregularly longitudinally rugose. Forecoxae unsculptured, smooth, and shining. Both waist segments with strong sculpture, mainly longitudinally rugose. Gaster unsculptured, smooth, and shining. Ground sculpture generally faint to absent everywhere on body. Whole body with abundant, very long, and fine standing hairs; first gastral tergite without appressed pubescence. Anterior edges of antennal scapes with suberect to erect hairs. Body of uniform very dark brown to black colour.

##### Distribution and ecology.

Currently, *Tetramorium capillosum* is found throughout Gabon, southern Cameroon, eastern Central African Republic, and then much further east in the northeastern Democratic Republic of Congo and northwestern Uganda (Figure 7). As noted above, the disjunctive range is almost certainly due to a sampling artefact, and it is very likely that *Tetramorium capillosum* is also encountered in Congo, and in much more of the Democratic Republic of Congo than just the northeastern locality of Epulu. Also, it seems unlikely that the species does not occur in Equatorial Guinea since it is found further north, south, and east, and was likely missed due to a sampling artefact. With the available material in mind, it seems that *Tetramorium capillosum* lives in leaf litter and/or on the ground, and is found at elevations from 40 to 1081 m.

##### Discussion.

Within the African *Tetramorium tortuosum* group *Tetramorium capillosum* is easily recognisable. *Tetramorium capillosum* differs from *Tetramorium hecate* and *Tetramorium tabarum* in overall body size and colouration. The latter two are smaller species (WL 0.69 - 0.83; PW 42 - 52) either bicoloured or of uniform brown colour, whereas *Tetramorium capillosum* is noticeably larger (WL 1.02 - 1.19; PW 60 - 68) and of a very dark brown to black colour. However, both body size and colouration are often variable within the genus *Tetramorium*. *Tetramorium capillosum* also differs from *Tetramorium hecate* in antennal scape length, eye size, and petiolar node shape. In *Tetramorium capillosum* the antennal scapes are moderately long (SI 80 - 83), eyes are of moderate size (OI 24 - 25), and the petiolar node has anterodorsal and posterodorsal margins that are relatively rounded, with the posterodorsal margin situated higher than the anterodorsal. In contrast, *Tetramorium hecate* scapes are relatively short (SI 73 - 77), eyes are relatively large (OI 27 - 31), and the petiolar node is rectangular nodiform with anterodorsal and posterodorsal angles sharply defined and at about the same height. Furthermore, *Tetramorium tabarum* significantly varies from *Tetramorium capillosum* in eye size, propodeal spine length, and sculpture on mandibles and postpetiole. *Tetramorium tabarum* has much larger eyes (OI 27 - 31), much shorter spines (PSLI 22 - 25), and the mandibles and the postpetiole are unsculptured, whereas *Tetramorium capillosum* has smaller eyes (OI 24 - 25), much longer spines (PSLI 31 - 43), and conspicuously sculptured mandibles and postpetiole.

It should be noted that despite a relatively broad distribution range from the Gulf of Guinea to northwest Uganda, *Tetramorium capillosum* remains morphologically remarkably stable without noticeable intraspecific variation.

#### Tetramorium hecate

Hita Garcia & Fisher sp. n.

urn:lsid:zoobank.org:act:BEEEF558-1DDA-40AB-8D79-597684B38033

http://species-id.net/wiki/Tetramorium_hecate

**Figure 4.**
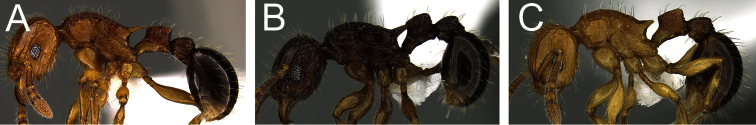
Variations of *Tetramorium hecate*. **A** bicoloured form with long propodeal spines (CASENT0248334) **B** uniform brown coloured form with long propodeal spines (CASENT0235157) **C** bicoloured form with short propodeal spines (CASENT0235154).

**Figure 5.**
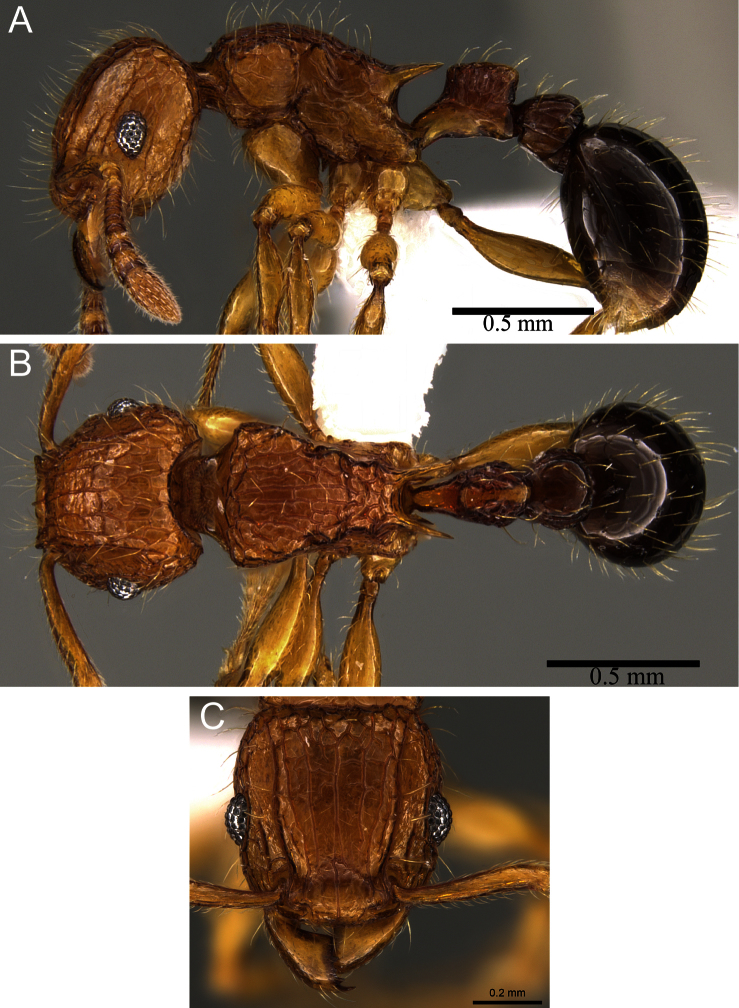
*Tetramorium hecate* holotype worker (CASENT0248334). **A** Body in profile **B** Body in dorsal view **C** head in full-face view.

Figures 1A , 4A, 4B, 4C , 5A, 5B, 5C , 7

##### Type material.

**Holotype**, pinned worker, GABON, Province Estuaire, F.C. Mondah, 21 km 331°NNW Libreville, 0°34.6'N, 9°20.1'E, 10 m, littoral rainforest, sifted litter (leaf mold, rotten wood), collection code BLF01742, 24.II.1998 (*B.L. Fisher*) [unique specimen identifier CASENT0248334] [CASC]. **Paratypes**, 16 pinned workers with same data as holotype [BMNH: CASENT0248332; CASC: CASENT0235154; CASENT0248333; CASENT0248335; CASENT0248336; CASENT0248337; CASENT0248338; CASENT0248339; CASENT0248340; CASENT0248341; CASENT0248342; MCZ: CASENT0248343; MHNG: CASENT0248344; NHMB: CASENT0248345].

##### Non-type material.

CAMEROON: Campo Reserve, 2°36'N, 9°56'E, 40 m, 25.X.1991 (*D.M. Olson*); Mbalmayo, XI.1993 (*N. Storck*); Nkoemvon, 16.III.1980 (*D. Jackson*); Sud, P.N. Campo, 43.3 km 108°ESE Campo, 2.2825N, 10.2062E, 290 m, rainforest, 7.IV.2000 (*B.L. Fisher*); Sud, Res. de Faune de Campo, 2.16 km 106°ESE Ébodjé, 2.5678N, 9.8443E, 10 m, littoral rainforest, 9.IV.2000 (*B.L. Fisher*); Sud, Res. de Faune de Campo, Massif des Mamelles, 15.1 km 84°E Ébodjé, 2.5942N, 9.9595E, 180 m, rainforest, 4.IV.2000 (*B.L. Fisher*); Sud-Ouest, Bimbia Forest, 7.4 km 119°ESE Limbe, 3.9818N, 9.2625E, 40 m, rainforest, 14.IV.2000 (*B.L. Fisher*); GABON: Province Estuaire, Mondah Forest, near Libreville, 3.XII.1987 (*J. Noyes*); Province Estuaire, F.C. Mondah, 21 km 331°NNW Libreville, 0°34.6'N, 9°20.1'E, 10 m, littoral rainforest, 24.II.1998 (*B.L. Fisher*).

##### Diagnosis.

*Tetramorium hecate* differs from the other species of the group by the following character combination: antennal scapes relatively short (SI 73 - 77); eyes large (OI 27 - 31); petiolar node rectangular nodiform with anterodorsal and posterodorsal margins strongly angulate and situated at about the same height; mandibles unsculptured, smooth, and shining; petiole and postpetiole usually with weak sculpture; body colouration ranging from uniformly brown to head, mesosoma, waist segments yellowish to bright orange contrasting with very dark brown to black gaster.

##### Worker measurements

**(N=12).** HL 0.58 - 0.67 (0.63); HW 0.54 - 0.64 (0.60); SL 0.41 - 0.49 (0.45); EL 0.15 - 0.20 (0.17); PH 0.28 - 0.36 (0.33); PW 0.42 - 0.52 (0.47); WL 0.69 - 0.83 (0.77); PSL 0.12 - 0.26 (0.20); PTL 0.20 - 0.26 (0.24); PTH 0.22 - 0.27 (0.25); PTW 0.17 - 0.22 (0.20); PPL 0.19 - 0.22 (0.21); PPH 0.22 - 0.28 (0.25); PPW 0.24 - 0.29 (0.27); CI 93 - 96 (94); SI 73 - 77 (76); OI 27 - 31 (28); DMI 58 - 63 (61); LMI 41 - 44 (42); PSLI 21 - 38 (32); PeNI 39 - 46 (43); LPeI 89 - 100 (94); DPeI 81 - 89 (84); PpNI 55 - 62 (57); LPpI 80 - 91 (86); DPpI 120 - 130 (126); PPI 129 - 142 (135).

##### Worker description.

Head longer than wide (CI 93 - 96); posterior head margin weakly concave. Anterior clypeal margin usually entire and convex, sometimes with very small median notch only visible under higher magnifications. Frontal carinae strongly developed, approaching or ending at posterior head margin. Antennal scrobes well developed, moderately shallow, and with clearly defined margins all around. Antennal scapes relatively short, not reaching posterior head margin (SI 73 - 77). Eyes large (OI 27 - 31). Mesosomal outline in profile weakly convex, moderately marginate from lateral to dorsal mesosoma; promesonotal suture and metanotal groove absent; mesosoma comparatively stout and high (LMI 41 - 44). Propodeal spines usually long to very long (PSLI 30 - 38), elongate-triangular to spinose, and acute, rarely spines reduced, short, elongate-triangular, and acute (PSLI 20 - 21); propodeal lobes short, triangular to elongate-triangular, and acute. Petiolar node in profile rectangular nodiform, approximately as high as long to weakly higher than long (LPeI 89 - 100), anterior and posterior faces approximately parallel, anterodorsal and posterodorsal margins situated at about the same height, anterodorsal and posterodorsal angles well-developed and rectangular, petiolar dorsum flat; node in dorsal view around 1.1 to 1.2 times longer than wide (DPeI 81 - 89). Postpetiole in profile globular to subglobular, approximately 1.1 to 1.2 times higher than long (LPpI 80 - 91); in dorsal view around 1.2 to 1.3 times wider than long (DPpI 120 - 130). Postpetiole in profile appearing less voluminous than petiolar node, in dorsal view approximately 1.3 to 1.4 times wider than petiolar node (PPI 129 - 142). Mandibles unsculptured, smooth, and shining; clypeus longitudinally rugulose, usually with three distinct rugulae, median rugula better developed than remainder, rugulae often with cross-meshes; cephalic dorsum between frontal carinae irregularly longitudinally rugose to reticulate rugose, posteriorly towards posterior head margin well reticulate-rugose, anteriorly towards posterior clypeal margin more regularly longitudinally rugose (usually with five to eight longitudinal rugae); scrobal area mostly unsculptured; lateral and ventral head longitudinally rugose to reticulate-rugose. Mesosoma laterally irregularly rugose, dorsally distinctly longitudinally rugose. Forecoxae unsculptured, smooth, and shining. Both waist segments laterally weakly, irregularly rugulose/rugose, dorsally mostly unsculptured, smooth, and shining. Postpetiole and gaster unsculptured, smooth, and shining. Ground sculpture generally faint to absent everywhere on body. Whole body with abundant, long, and fine standing hairs; first gastral tergite without appressed pubescence. Anterior edges of antennal scapes with suberect to erect hairs. Body colouration relatively variable, ranging from bicoloured with head, mesosoma, legs, and waist segments yellowish to bright orange contrasting with very dark brown to black gaster to whole body uniformly brown.

##### Etymology.

The name of the new species is inspired by the ancient Latin and Greek goddess “Hecate” or “Hekate”, also known as the “triple Hecate” or “three-faced Hecate”, and refers to the morphological variation in colouration and propodeal spine length observed in *Tetramorium hecate*. The species epithet is a nominative noun in apposition, and thus invariant.

##### Distribution and ecology.

The new species is currently only known from Cameroon and Gabon (Figure 7). All localities are situated at relatively low elevations ranging from 10 to 300 m, and most are either rainforests or littoral rainforests. Most of the available material was collected through litter sifting, which suggests that the preferred microhabitat of *Tetramorium hecate* is forest leaf litter.

##### Discussion.

*Tetramorium hecate* is unlikely to be misidentified with the other two species of the group. The most obvious difference between *Tetramorium hecate* and *Tetramorium capillosum* and *Tetramorium tabarum* is the shape of the petiolar node. In the latter two the node is nodiform with relatively rounded anterodorsal and posterodorsal margins, and, in addition, the posterodorsal margin is situated noticeably higher than the anterodorsal margin. In contrast, the node of *Tetramorium hecate* is nodiform, with clearly rectangular anterodorsal and posterodorsal margins, situated at about the same height. The second-most important character is antennal scape length. *Tetramorium hecate* has the shortest scapes of the three species (SI 73 - 77), strongly contrasting with the longer scapes of the other two species (SI 80 - 86). Furthermore, *Tetramorium hecate* is also a much smaller species with much larger eyes and very different colouration than *Tetramorium capillosum* (see description of the latter for more details). The very well developed antennal scrobes with clearly defined margins all around also separate *Tetramorium hecate* from *Tetramorium tabarum* since the scrobes of the latter are shallow and without posterior and ventral margins.

The new species shows an intriguing variation in colouration and propodeal spine length. At the initial sorting stage of this revision, we considered the material listed here as *Tetramorium hecate* to consist of three potential new species: a strongly bicoloured one with long propodeal spines (Figure 4A), a uniformly brown coloured one with long propodeal spines (Figure 4B), and a bicoloured one with shorter spines (Figure 4C). However, after examination of all the material, it became apparent that they belong to just one species having a moderate variation in colour, which includes a few specimens with exceptionally short propodeal spines. The differences in colouration appear distinct at first when looking at few specimens, but the examination of several hundred specimens showed that there is a gradual variation that ranges from strongly bicoloured to completely uniformly coloured with more than half of the specimens being intermediate. Generally, the spines are long to very long (PSLI 30 - 38) throughout the examined material of several hundred specimens, the exception being three specimens with very short propodeal spines (PSLI 20 -21). Apart from the spine length, however, no single character would justify a separation of these specimens from the main material. Consequently, we are very confident that all the material examined belongs to just one species.

#### Tetramorium tabarum

Bolton

http://species-id.net/wiki/Tetramorium_tabarum

**Figure 6.**
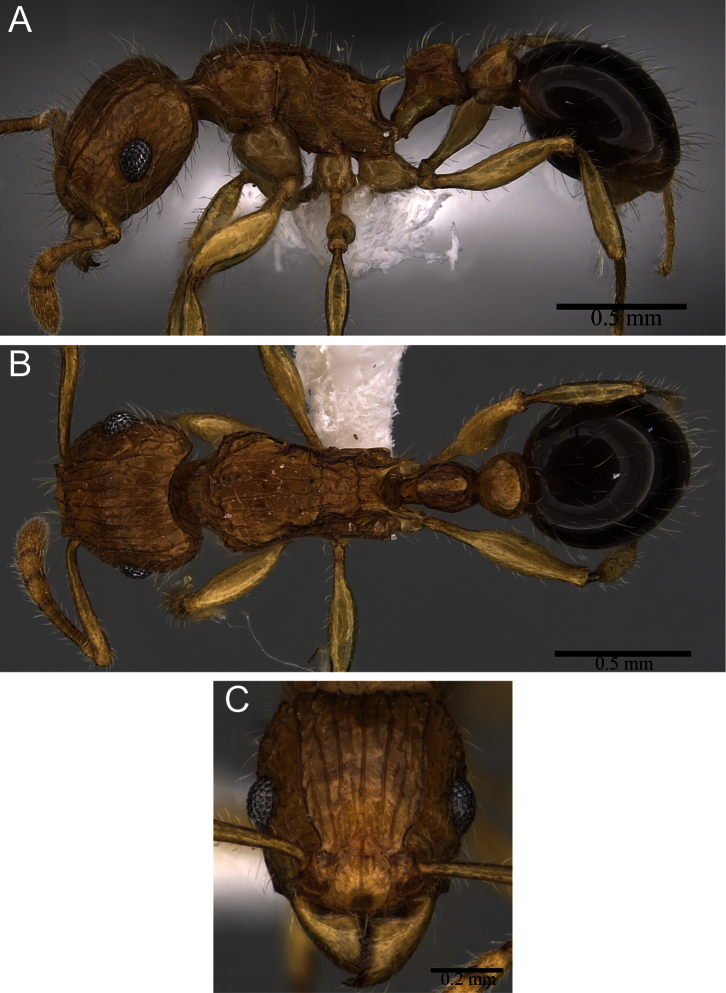
*Tetramorium tabarum* worker (CASENT0316967). **A** Body in profile **B** Body in dorsal view **C** head in full-face view.

**Figure 7.**
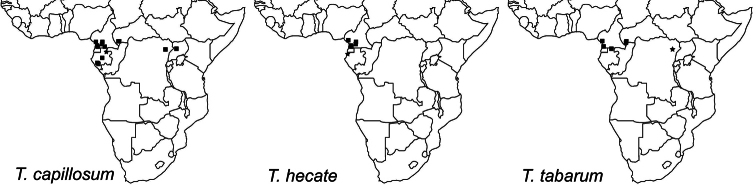
Geographic distribution maps for the species treated in this study. Star symbols represent type localities while rectangles represent non-type localities.

Figures 1C , 2B, 2D , 6A, 6B, 6C , 7

Tetramorium tabarum Bolton, 1980: 236.

##### Type material.

Holotype, pinned worker, DEMOCRATIC REPUBLIC OF CONGO, Epulu, 4.I.1949 (*J.C. Bradley*) [MCZ] [examined].

##### Non-type material.

CAMEROON: Mvini, 21.XII.1988 (*A. Dejean*); Sud-Ouest, Bimbia Forest, 7.4 km 119°ESE Limbe, 3.9818N, 9.2625E, 40 m, rainforest, 14.IV.2000 (*B.L. Fisher*); CENTRALAFRICANREPUBLIC: Prefecture Sangha-Mbaéré, Réserve Spéciale de Forêt Dense de Dzanga-Sangha, 12.7 km 326°NW Bayanga, 3.005N, 16.1933E, 420 m, rainforest, 10.–17.V.2001 (*B.L. Fisher*); Prefecture Sangha-Mbaéré, Parc National Dzanga-Ndoki, 37.9 km 169°S Lidjombo, 2.3707N, 16.1725E, 360 m, rainforest, 20.–28.V.2001 (*B.L. Fisher*); GABON: Woleu-Ntem, 31.3 km 108°ESE Minvoul, 2.08N, 12.4067E, 600 m, rainforest, 17.II.1998 (*B.L. Fisher*).

##### Diagnosis.

*Tetramorium tabarum* is easily recognisable within the Afrotropical *Tetramorium tortuosum* group by the following character combination: antennal scape moderately long (SI 84 - 86); eyes large (OI 27 - 31); petiolar node nodiform with anterodorsal and posterodorsal margins relatively rounded, posterodorsal margin situated higher than anterodorsal margin, dorsum convex; mandibles unsculptured, smooth, and shining; petiole with very weak sculpture and postpetiole completely unsculptured; head, mesosoma, waist segments yellowish to bright orange, gaster very dark brown to black.

##### Worker measurements

**(N=10).** HL 0.61 - 0.66 (0.63); HW 0.55 - 0.60 (0.57); SL 0.47 - 0.52 (0.49); EL 0.16 - 0.18 (0.17); PH 0.30 - 0.34 (0.32); PW 0.43 - 0.46 (0.44); WL 0.75 - 0.82 (0.79); PSL 0.14 - 0.16 (0.15); PTL 0.20 - 0.22 (0.20); PTH 0.24 - 0.27 (0.25); PTW 0.16 - 0.18 (0.17); PPL 0.19 - 0.21 (0.20); PPH 0.23 - 0.27 (0.25); PPW 0.24 - 0.27 (0.26); CI 90 - 92 (91); SI 84 - 86 (85); OI 27 - 31 (29); DMI 55 - 58 (56); LMI 38 - 43 (41); PSLI 22 - 25 (23); PeNI 37 - 39 (38); LPeI 78 - 82 (81); DPeI 80 - 85 (82); PpNI 56 - 59 (58); LPpI 79 - 85 (82); DPpI 124 - 130 (127); PPI 147 - 156 (152).

##### Worker description.

Head significantly longer than wide (CI 90 - 92); posterior head margin moderately concave. Anterior clypeal margin entire and convex. Frontal carinae strongly developed, approaching or ending at posterior head margin. Antennal scrobes developed, but shallow and without clearly defined posterior and ventral margins. Antennal scapes moderately long, not reaching posterior head margin (SI 84 - 86). Eyes large (OI 27 - 31). Mesosomal outline in profile weakly convex, moderately marginate from lateral to dorsal mesosoma; promesonotal suture and metanotal groove absent; mesosoma comparatively stout and high (LMI 38 - 43). Propodeal spines relatively short to medium-sized, elongate-triangular to spinose, and acute (PSLI 22 - 25); propodeal lobes short, triangular to elongate-triangular, and acute. Petiolar node in profile rectangular nodiform, approximately 1.2 to 1.3 times higher than long (LPeI 78 - 82), anterior and posterior faces approximately parallel, posterodorsal margin situated higher than anterodorsal, anterodorsal and posterodorsal angles relatively rounded, petiolar dorsum convex; node in dorsal view approximately 1.2 times longer than wide (DPeI 80 - 85). Postpetiole in profile subglobular and moderately anteroposteriorly compressed, approximately 1.2 to 1.3 times higher than long (LPpI 79 - 85); in dorsal view around 1.2 to 1.3 times wider than long (DPpI 124 - 130). Postpetiole in profile appearing less voluminous than petiolar node, in dorsal view approximately 1.5 to 1.6 times wider than petiolar node (PPI 147 - 156). Mandibles unsculptured, smooth, and shining; clypeus longitudinally rugulose, usually with three rugulae, median rugula better developed; cephalic dorsum between frontal carinae with five to eight longitudinal rugae, most rugae running unbroken from posterior clypeal margin to posterior head margin, few rugae interrupted, but none with cross-meshes; scrobal area mostly unsculptured; lateral and ventral head longitudinally rugose to reticulate-rugose. Mesosoma laterally irregularly rugose, dorsally distinctly longitudinally rugose. Forecoxae unsculptured, smooth, and shining. Petiolar node laterally weakly to moderately longitudinally rugose. Postpetiole and gaster unsculptured, smooth, and shining. Ground sculpture generally faint to absent everywhere on body. Whole body with abundant, long, and fine standing hairs; first gastral tergite without appressed pubescence. Anterior edges of antennal scapes with suberect to erect hairs. Head, mesosoma, legs, and waist segments yellowish to bright orange, contrasting with very dark brown to black gaster.

##### Distribution and ecology.

*Tetramorium tabarum* is known to occur in northern Gabon, western Cameroon close to the Gulf of Guinea, the southwest of the Central African Republic, and from the type locality in the northeast of the Democratic Republic of Congo (Figure 7). Its distribution seems even more disjunctive than that of *Tetramorium capillosum*, but is very likely due to a sampling artefact as already noted above for the latter species. We are very confident that more material of *Tetramorium tabarum* will be sampled in the area between the known localities. This species appears to be the rarest in the complex with far less material available than for the other two species. One reason for this scarcity might be its preference for a different microhabitat. The latter two were mainly sampled from the leaf litter/ground, whereas most specimens of *Tetramorium tabarum* were collected from vegetation. Additional sampling on low vegetation or canopy might yield more material.

## Discussion

As already pointed out in the above descriptions of the other two species, *Tetramorium tabarum* is easily recognisable within the complex. It differs significantly from *Tetramorium capillosum* in eye size (OI 27 - 31 vs. OI 24 - 25), propodeal spine length (PSLI 22 - 25 vs. PSLI 31 - 43), and sculpture on mandibles and postpetiole, which is present and conspicuous in *Tetramorium capillosum* while absent in *Tetramorium tabarum*. The latter is also much smaller (WL 0.75 - 0.82) and bicoloured with a dark brown to black gaster contrasting with the remaining yellowish to orange body, which contrasts with the larger size (WL 1.02 - 1.19) and the uniformly very dark brown to black colouration of *Tetramorium capillosum*. Despite being often also bicoloured and within the same morphometric range, *Tetramorium hecate* is unlikely to be confused with *Tetramorium tabarum*. The antennal scapes are significantly longer in *Tetramorium tabarum* (SI 84 - 86) than in *Tetramorium hecate* (SI 73 - 77). More importantly, the petiolar node of *Tetramorium tabarum* has relatively rounded anterodorsal and posterodorsal margins with the posterodorsal margin situated higher than the anterodorsal, and the dorsum is convex, whereas in *Tetramorium hecate* the anterodorsal and posterodorsal margins are sharply defined and at about the same height. The varying development of the antennal scrobes is another difference. *Tetramorium hecate* possesses very well-developed scrobes with margins all around while the scrobes of *Tetramorium tabarum* are shallow without clear posterior and ventral margins.

The material currently available for *Tetramorium tabarum* is relatively limited, but it seems that intraspecific variation is very low.

## Supplementary Material

XML Treatment for Tetramorium capillosum

XML Treatment for Tetramorium hecate

XML Treatment for Tetramorium tabarum
